# Long-range temporal correlations in resting state alpha oscillations in major depressive disorder and obsessive-compulsive disorder

**DOI:** 10.3389/fninf.2024.1339590

**Published:** 2024-02-21

**Authors:** Ekaterina Proshina, Olga Martynova, Galina Portnova, Guzal Khayrullina, Olga Sysoeva

**Affiliations:** ^1^Centre for Cognition and Decision Making, Institute for Cognitive Neuroscience, National Research University Higher School of Economics, Moscow, Russia; ^2^Laboratory of Human Higher Nervous Activity, Institute of Higher Nervous Activity and Neurophysiology of RAS, Moscow, Russia; ^3^Faculty of Biology and Biotechnology, National Research University Higher School of Economics, Moscow, Russia; ^4^Sirius Center for Cognitive Sciences, Sirius University of Science and Technology, Sochi, Russia

**Keywords:** obsessive-compulsive disorder, major depressive disorder, electroencephalography, detrended fluctuation analysis, long-range temporal correlations, resting state

## Abstract

**Introduction:**

Mental disorders are a significant concern in contemporary society, with a pressing need to identify biological markers. Long-range temporal correlations (LRTC) of brain rhythms have been widespread in clinical cohort studies, especially in major depressive disorder (MDD). However, research on LRTC in obsessive-compulsive disorder (OCD) is severely limited. Given the high co-occurrence of OCD and MDD, we conducted a comparative LRTC investigation. We assumed that the LRTC patterns will allow us to compare measures of brain cortical balance of excitation and inhibition in OCD and MDD, which will be useful in the area of differential diagnosis.

**Methods:**

In this study, we used the 64-channel resting state EEG of 29 MDD participants, 26 OCD participants, and a control group of 37 volunteers. Detrended fluctuation analyzes was used to assess LRTC.

**Results:**

Our results indicate that all scaling exponents of the three subject groups exhibited persistent LRTC of EEG oscillations. There was a tendency for LRTC to be higher in disorders than in controls, but statistically significant differences were found between the OCD and control groups in the entire frontal and left parietal occipital areas, and between the MDD and OCD groups in the middle and right frontal areas.

**Discussion:**

We believe that these results indicate abnormalities in the inhibitory and excitatory neurotransmitter systems, predominantly affecting areas related to executive functions.

## 1 Introduction

Obsessive-compulsive disorder (OCD) and major depressive disorder (MDD) are common mental disorders that can significantly impact a person’s quality of life. These two conditions manifest differently: MDD is associated with anhedonia, persistent feelings of sadness, hopelessness, and loss of interest, while OCD is characterized by intrusive thoughts that cause distress (obsessions) and repetitive actions performed to reduce distress (compulsions) (American Psychiatric Association, 2013). However, studies have shown that 17–60% of people diagnosed with OCD have comorbid depression (Overbeek et al., 2002). In addition, both conditions are often characterized by anxiety. It was discovered that 45.7% of respondents with lifetime MDD had one or more lifetime anxiety disorders (Kessler et al., 2015). Feelings of anxiety related to unwanted and uncomfortable thoughts and images are among the most common among people with OCD. Since OCD and MDD are often co-occurring disorders and their symptoms occur simultaneously, it is important to find an objective marker that could potentially be used in the differential diagnosis.

It is assumed that objective measures such as the resting-state electroencephalogram (EEG) may be a useful tool in this regard. Most often, studies are conducted on the frequency characteristics of the EEG signal, as well as the strength of connections at the level of sensors or cortical sources. Regarding the power frequency in MDD, the predictive role of multiple bands is mentioned: increased gamma power (Fitzgerald and Watson, 2018), reduced alpha-2 power over central regions of the left hemisphere (Lee et al., 2018), global decreased alpha power (Price et al., 2008), and conversely increased alpha power (Kemp et al., 2010; Jaworska et al., 2012). Grin-Yatsenko et al. (2010) found increased activity in theta, alpha, and beta bands at the occipital and parietal areas in depression. The authors assumed that an increase in slow EEG activity may reflect a decreased cortical activation in these brain regions (Grin-Yatsenko et al., 2010). Regarding the strength of connections, recent meta-analyzes of 52 studies have reported no differences in EEG functional connectivity in the delta and gamma frequencies between depression and control groups. The most pronounced differences were observed in alpha, theta, and beta bands (Miljevic et al., 2023). However, difficulties in reproducible conclusions are highlighted due to significant inconsistencies between the study design and methodology (Miljevic et al., 2023).

Regarding the resting-state EEG characteristic in OCD, a recent meta-analysis has shown that the most prominent changes are the power increases at delta and theta frequencies and decreases at alpha, beta, and gamma frequencies (Newson and Thiagarajan, 2019). In addition, a change in EEG graph metrics was detected in OCD compared to controls, suggesting a disruption in information processing in the brain (Tan et al., 2019, 2022). Decreased non-linear coherence was found in OCD for the beta frequency range for connectivity measures between frontal brain areas (the anterior cingulate cortex, the superior frontal gyrus, and the left medial frontal gyrus; Olbrich et al., 2013).

In recent years, the analysis of the nonlinear characteristics of the signal has been developing, which allows for considering the non-stationarity of the biological brain signal. The markers of mental disorders can be reflected in the temporal features of information processing in bioelectrical brain activity. The ability to integrate information over extended periods of time can be assessed with long-range temporal correlations (LRTCs) of the EEG signal. It is assumed that LRTCs indicate the presence of a scale-free structure of neuronal activation on multiple time scales that is important for optimal neuronal processing in the human brain (Linkenkaer-Hansen et al., 2001; Hardstone et al., 2012; Palva et al., 2013). The LRTCs reflect the self-affinity of the EEG signal, which is a non-stationary stochastic process. Most of the articles devoted to the study of LRTCs consider the alpha frequency range as it has pronounced oscillatory patterns in the spectra of ongoing EEG/MEG (Linkenkaer-Hansen et al., 2001; Nikulin and Brismar, 2004, 2005). The normalization of alpha LRTCs, which was associated with symptom relief, was found after neurofeedback training in posttraumatic stress disorder patients (Ros et al., 2016).

LRTC is considered a measure of excitation/inhibition balance in neural networks (Bruining et al., 2020; Ahmad et al., 2022). The studies suggest that both OCD and MDD are associated with altered cortical excitation and inhibition, which may contribute to the symptoms of these disorders (Richter et al., 2012; Kang et al., 2019; Rodrigues Da Silva et al., 2022). Specifically, individuals diagnosed with OCD may have an inability to inhibit unwanted intrusive thoughts, while individuals with MDD may experience reduced inhibition that impairs stimulus processing. Nevertheless, we found no comparative studies on EEG LRTC in OCD and MDD patients or controls. Although one study has shown measurements of inhibition and excitation in OCD, MDD, and schizophrenia using transcranial magnetic stimulation (TMS), it found inhibitory deficits in all three conditions and specific enhancement of intracortical facilitations in OCD (Radhu et al., 2013).

In this study, we investigated the intrinsic brain activity during rest with closed eyes in groups of individuals diagnosed with MDD, OCD, and healthy controls. The LRTCs were computed by means of detrended fluctuation analyzes (DFA; Peng et al., 1995). DFA produces estimates of the magnitude of detrended fluctuations at different scales (windows). Using DFA, the fractal scaling exponent (α) was calculated, which allows us to evaluate the self-similarity in the time series. We assume that the LRTC patterns will allow us to compare measures of brain cortical balance of excitation and inhibition in OCD and MDD, which will be useful in the area of differential diagnosis.

## 2 Materials and methods

### 2.1 Participants

We examined the EEG data of three samples: 29 (25 females, mean age 36.6, SD = 10) participants with MDD, 26 participants with OCD (17 females, mean age 24.8, SD = 6.1), and a control group of 37 volunteers (31 females, mean age 28.2, SD = 11.1). The control group comprised participants who reported the absence of neurological or psychiatric disorders, major medical disorders, sustained head injuries, alcohol or drug abuse, or current treatment with vasoactive or psychotropic medication. All applicable subject protection guidelines and regulations were followed in the conduct of the research in accordance with the Declaration of Helsinki. The study protocol was approved by the ethical committee of the Institute of Higher Nervous Activity and Neurophysiology of the Russian Academy of Sciences (Ethics protocol: No. 2, 30 April 2021, Ethics protocol: No. 1, 25 February 2021). All participants signed a written informed consent.

### 2.2 Measurement of major depressive disorder

Volunteers with MDD were recruited for the study following clinical examinations at partner hospitals. All participants were assessed by a psychiatrist and had a documented history of recurrent depression, indicating multiple depressive episodes throughout their lives. A subset of subjects received maintenance doses of antidepressant medication during the study period, as detailed in Table 1. Before the EEG recording, each individual completed the Beck Depression Inventory (BDI) (Beck et al., 1961), a widely recognized screening tool in clinical practice and research studies.

**Table 1:** Medications taken by members of the MDD and OCD group.

Medication	Number of participants
*MDD group*	
Selective serotonin reuptake inhibitors (antidepressants)	4
Serotonin 5-HT2C Receptor Antagonists (antidepressants)	1
*OCD group*	
Selective serotonin reuptake inhibitors (antidepressants)	15
Selective serotonin reuptake inhibitors (antidepressants) + anticonvulsants	4
Antipsychotics + anticonvulsants	2

### 2.3 Measurement of obsessive-compulsive disorder

The participants with diagnosed OCD were recruited by the clinical psychologist (G.K.). A structured interview was conducted to confirm that patients met the criteria set by the International Statistical Classification of Diseases and Related Health Problems 10th Revision (Brämer, 1988). The Yale-Brown Obsessive Compulsive Scale (Y-BOCS) (Goodman et al., 1989) was employed to measure the severity of their condition. The scale, commonly used by clinicians, provides five rating dimensions for obsessions and compulsions: time spent or occupied, interference with functioning or relationships, degree of distress, resistance, and control. The Y-BOCS consists of 10 items, and the total score enables an assessment of overall severity. In the presented sample, the mean test score was 20.9 ± 8.27. Scores on the measure range from 0 to 40, with 0–7 indicating subclinical symptoms, 8–15 indicating mild symptoms, 16–23 indicating moderate symptoms, 24–31 indicating severe symptoms, and 32–40 indicating extreme symptoms. Control participants’ scores on the Y-BOCS are likely to be closer to the lower end of the range. Patients with psychotic disorders and atypical or reactive depressive episodes were excluded from the study, as were those who had traumatic brain injuries, neurological disorders, structural abnormalities and uncorrected vision. Table 1 displays the medication taken by individuals in the MDD and OCD groups.

### 2.4 EEG data acquisition

EEG recording was performed in a soundproof, dimly illuminated room, participants were asked to minimize movements. The procedure consisted of four 30-s recordings, two with eyes closed and two with eyes open, alternating sequentially. It is hypothesized that a resting state paradigm with alternating periods of open and closed eyes is optimal for studying the electroencephalographic correlates of mental processes inherent in humans. It allows a moderate level of arousal to be maintained (Gale, 1983). 63 EEG electrodes were distributed according to the international 10–10 electrode placement system. A BrainProduct amplifier with a 0.1–100-Hz analog bandpass filter was used for signal amplification. The sampling rate was 500 Hz. Electrode impedances were kept at or below 15 kilo-ohms. The frontal electrode was used as the ground and Cz as the reference. In this work, we have focused solely on studying the resting state with eyes closed, an approach that allows for the assessment of intrinsic brain activity while minimizing the influence of external interruptions. This approach has been used in numerous studies where, although the eyes were alternately closed and open to prevent drowsiness during the closed-eye periods (Maltez et al., 2004), only the closed-eye portions of the recording were used for analysis (Nikulin and Brismar, 2005; Nikulin et al., 2012). Therefore, only two eye-closed segments of the resting state EEG were further analyzed.

### 2.5 EEG preprocessing

The EEG signal was filtered with an FIR filter in the range 1–30 Hz. The data was visually inspected for “bad” channels and re-referenced to the average reference. The data was downsampled to 100 Hz. Artifacts related to eye movements, blinking, and cardiac activity were removed using Independent Component Analysis (ICA).

**Figure 1:**
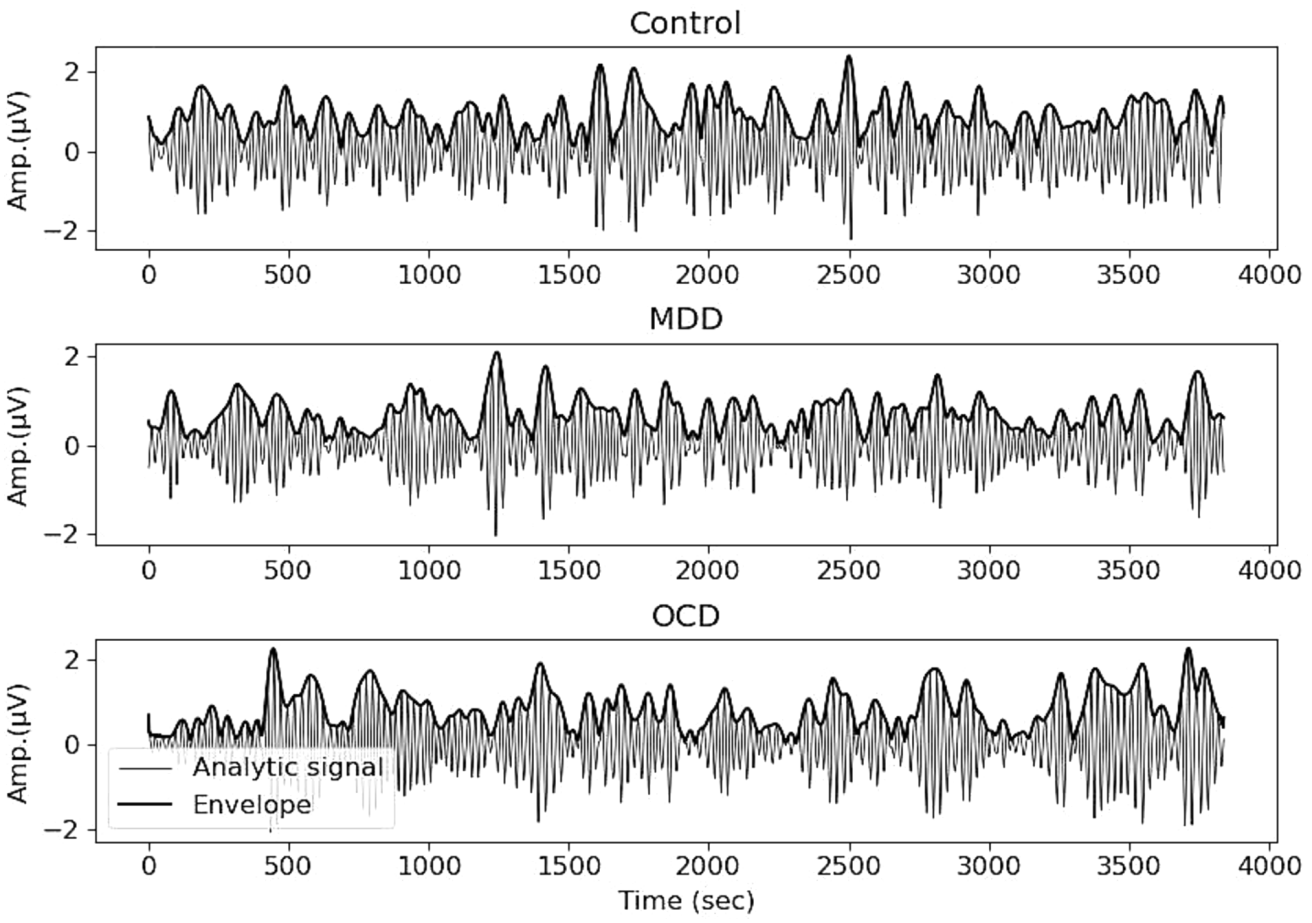
EEG recording, filtered in the range of 8–13 Hz, and the envelope (bold line) obtained with the Hilbert transform. Averages for control (top), MDD (middle) and OCD (bottom) groups, Electrode Pz.

As we focused on the alpha frequency range, we filtered the signal between 8 and 13 Hz using a Hamming window with 0.0194 passband ripple and 53 dB stopband attenuation. We divided the EEG signal into four epochs of 15 s each and followed the procedures outlined in Linkenkaer-Hansen et al. (2005). The analytic signal was computed using the Hilbert transform. Subsequently, the envelope was obtained by taking absolute values (Figure 1). All preprocessing was performed in MNE-Python.

### 2.6 Detrended fluctuation analyzes

The DFA method was applied to the envelope. A time series was integrated to create a cumulative sum signal. The integrated signal was divided into ten windows, with 50% overlap, logarithmically distributed between 0.6 and 3.5 s. A polynomial approximation was used to eliminate the local trend, and the root-mean-square fluctuation was calculated for each segment. Finally, a scaling pattern was derived by analyzing the relationship between fluctuation and segment length. The fractal scaling exponent (α) was calculated by determining the slope of the trend in the function of fluctuation intensity means in comparison to window sizes on a logarithmic scale (Peng et al., 1995; Hardstone et al., 2012). A α value of 0.5 is considered typical for white noise. The range of 0.5–1 characterizes the persistent long-range temporal correlations, where larger exponents indicate a slower decaying autocorrelation. The Python Nolds package was utilized for calculations (Schölzel, 2020). Furthermore, we estimated the goodness of fit for each α to understand how well it described the data. The goodness of fit was calculated as the squared correlation coefficient (*R*^2^). The mean *R*^2^ for each region was >0.95, which means it described the data well.

### 2.7 Statistical analysis

The α obtained from 63 electrodes were averaged over four 15-s epochs and over the regions of interest (ROI): left frontal (Fp1, AF3, AF7, F1, F5, F3, F7, FT7, FC5, FC3, FC1), right frontal (FP2, AF4, AF8, F2, F4, F6, F8, F8, FC2, FC4, FC6, FT8), middle frontal (AFz, Fz), left temporal (T7, TP7, CP5, C5), right temporal (T8, TP8, C6, CP6), left central parietal (C1, C3, CP3, CP1), right central parietal (C2, C4, CP2, CP4), middle central parietal (CPz, Pz), left parietal occipital (P1, P3, P5, P7, PO3, PO7, O1), right parietal occipital (P2, P4, P6, P8, PO4, PO8, O2), middle parietal occipital (POz, Oz). Our ROIs are based on the electrode position. EEG measures the electrical activity of large, synchronously firing populations of neurons in the brain with electrodes placed on the scalp. However, caution should be exercised when interpreting EEG activity in specific electrodes as representative of the activity of a given brain region due to its poor spatial resolution.

A mixed repeated measures ANOVA with one within-subjects (“ROI”) factor (11 levels) and one between-subjects (“group”) factor was used. Calculations were executed within the R software environment using the “rstatix” package. The Bonferroni test was used for a multiple-comparison correction. We also examined if there were differences in the severity of depression symptoms between the MDD and OCD groups, and if the α showed a correlation with the severity of MDD and OCD symptoms measured using the BDI and Y-BOCS, respectively.

## 3 Results

The MDD and OCD groups showed no significant differences on the BDI test scores (*t* = 0.955, df = 53, *p*-value = 0.343). The scores of the control group were significantly lower than in both the MDD (*t* = 8.045, df = 74, *p*-value <0.001) and OCD (*t* = −6.412, df = 66, *p*-value <0.001) groups. The mean and the standard deviation of BDI test scores by groups are: MDD—23 ± 10.1, OCD—20.2 ± 11.4, control—7.3 ± 5.1. No significant correlations were found between α and symptoms of OCD and MDD.

The mean age of participants in the MDD group (mean age = 36.6) was significantly higher than that of participants in the OCD group (mean age = 24.8), as demonstrated by statistical analysis (*T* (53) = 5.235, *p* < 0.001). No significant differences in age were found between the OCD and controls (mean age—28.2) (*p* = 0.15). The age of participants diagnosed with MDD was significantly higher than that of the control group, *T* (64) = 3.204, *p* = 0.002.

All scaling exponents of the three group subjects were in the interval of 0.6–1.1, indicating persistent LRTC of EEG oscillations in the alpha band. Averaged fractal scaling exponents, categorized by areas of interest and groups, are presented in Table 2. Mauchly’s test indicated that the assumption of sphericity had been violated (*p* < 0.001), therefore we applied the Greenhouse–Geisser (GG) correction (ε = 0.571) to produce a more valid critical *F*-value. The results of a mixed repeated measures ANOVA with GG correction (summarized in Table 3) indicate that the main effect of the “group” was significant (*F* (2, 89) = 3.14, *p* = 0.048), suggesting that a difference exists in the fractal scaling exponent between the groups of OCD, MDD, and healthy controls.

**Table 2:** Fractal scaling exponent (*α*) averaged by groups and areas.

ROIs	Control	MDD	OCD
Left central parietal	0.83	0.83	0.86
Right central parietal	0.84	0.82	0.85
Middle central parietal	0.87	0.86	0.90
Left parietal occipital	0.84	0.88	0.92
Right parietal occipital	0.87	0.89	0.92
Middle parietal occipital	0.87	0.88	0.94
Left temporal	0.79	0.82	0.83
Right temporal	0.81	0.84	0.84
Left frontal	0.79	0.81	0.87
Right frontal	0.78	0.81	0.88
Middle frontal	0.77	0.81	0.88

**Table 3:** ANOVA table for differences in fractal scaling exponent (*α*) between groups.

Effect	DFn	DFd	*F*	*p*	*p* < 0.05	GES
Group	2.00	89.00	3.140	0.048	*	0.048
ROI	5.71	508.28	26.757	< 0.001	*	0.079
Group:ROI	11.42	508.28	3.129	< 0.001	*	0.020

^*^GES, Greenhouse–Geisser correction.

**Figure 2:**
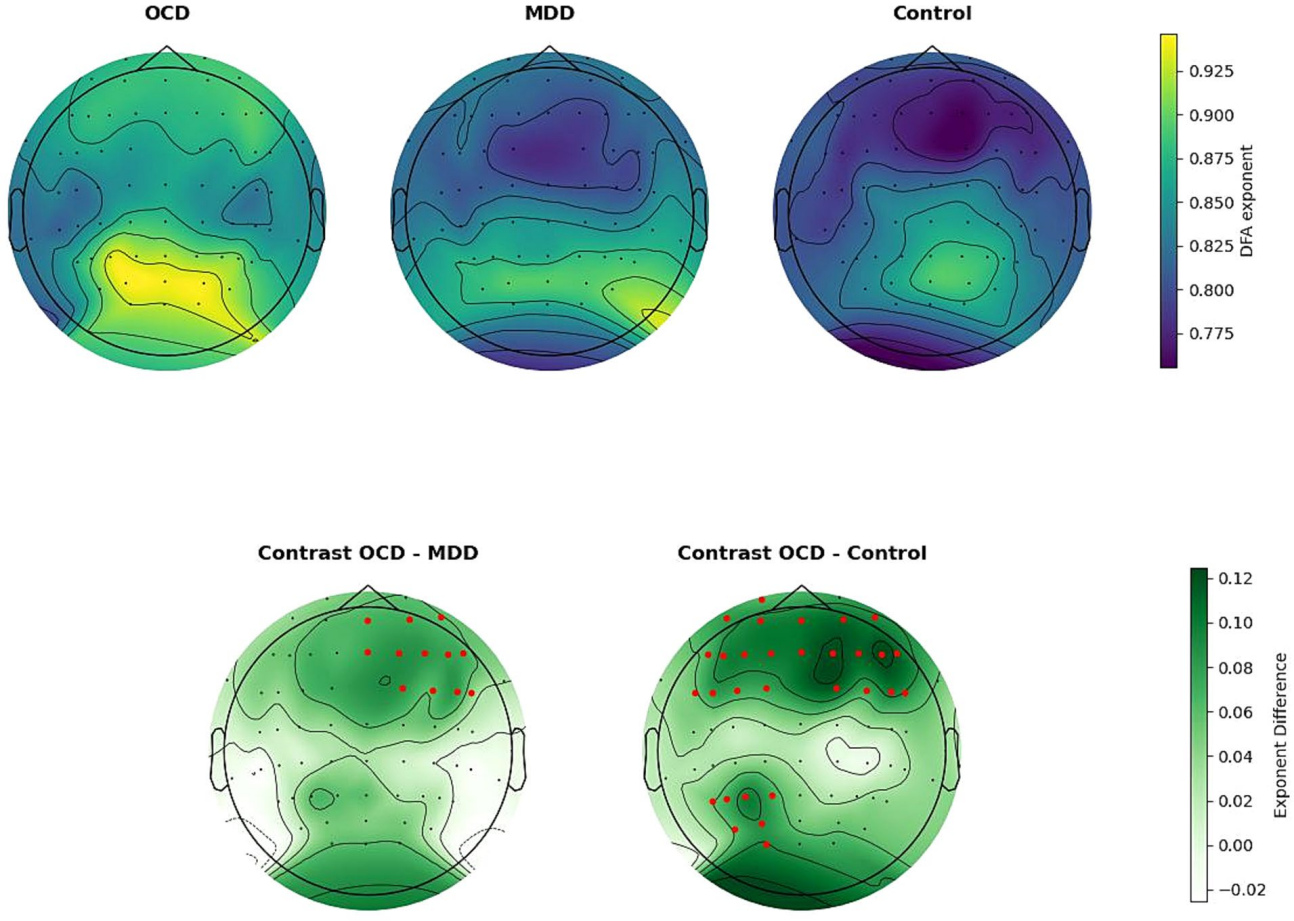
Upper line: spatial distribution of DFA exponents, averaged by group. Bottom line: group differences in DFA exponents. Electrodes are indicated by black dots. Electrodes included in the ROI that differ significantly between groups are indicated by the color red. The OCD DFA exponents were significantly higher than in the control group in the left frontal, middle frontal, right frontal, and left parietal occipital regions. Compared to the MDD group, DFA exponents in OCD were higher, in the middle frontal and right frontal regions.

The main effect of ROI with GG correction was not significant (*F* (5.71, 508.28) = 26.757, *p* = 0.079). The interaction effect between group and ROI was significant (*F* (11.42, 508.28) = 3.129, *p* = 0.02), indicating that there were statistically significant differences between the groups in certain areas of the brain. Post-hoc Bonferroni tests (results are summarized in Table 4) have revealed that the OCD group had a higher *α* score compared to the MDD and control groups. Specifically for OCD, the α score was higher than in the control group in the frontal regions: left frontal (*t* (979) = −3.164, *p*.adj = 0.004), middle frontal (*t* (979) = −3.972, *p*.adj < 0.001), right frontal (*t* (979) = −4.034, *p*.adj < 0.001), and in the left parietal occipital region (*t* (979) = −2.961, *p*.adj < 0.009). For MDD, the OCD α scores were higher in the middle frontal (t (979) = −2.607, *p*.adj = 0.027) and right frontal (*t* (979) = −2.632, *p*.adj = 0.025) regions (Figure 2).

**Table 4:** Differences between groups by ROIs, Bonferroni corrected.

ROI	Group 1	Group 2	df	Statistic	*p*	*p*.adj
left frontal	Control	OCD	979	–3.164	0.001	0.004
middle frontal	Control	OCD	979	–3.972	< 0.001	< 0.001
right frontal	Control	OCD	979	–4.034	< 0.001	< 0.001
left parietal occipital	Control	OCD	979	–2.961	0.003	0.009
middle frontal	MDD	OCD	979	−2.607	0.009	0.027
right frontal	MDD	OCD	979	–2.632	0.008	0.025

This study included participants who did not exhibit acute effects. Participants with OCD were taking medications on a long-term basis. The medication dosages for the MDD group were the lowest maintenance dosages. However, we cannot completely rule out the possibility of psychotropic drugs affecting the EEG. To address this concern, we conducted an additional group comparison using only patients who are currently not taking medication, although this resulted in a very small sample size (5 for OCD). After excluding those taking medication, the MDD group consisted of 23 participants.

The results of a mixed repeated measures ANOVA with GG correction indicate that the main effect of the “group” was significant (*F* (2, 62) = 1.22, *p* = 0.026), suggesting that a difference exists in the fractal scaling exponent between the groups of OCD, MDD, and healthy controls. The main effect of ROI with GG correction was not significant (*F* (5.05, 312.99) = 10.684, *p* = 0.054). The interaction effect between group and ROI was significant (*F* (10.10, 312.99) = 2.198, *p* = 0.023), indicating that there were statistically significant differences between the groups in certain areas of the brain. This difference persisted even when participants taking medication were excluded. Post-hoc Bonferroni tests have revealed that the OCD group had a higher α score compared to the control group at the middle frontal area (*t* (682) = −2.461, *p*.adj = 0.042) and right frontal area (*t* (682) = −2.642, *p*.adj = 0.025).

## 4 Discussion

We examined LRTCs in the amplitude fluctuations of ongoing neuronal oscillations in 29 participants with MDD, 26 participants with OCD, and 37 control subjects. We used DFA to assess the correlation properties of the time series, with a focus on identifying differences in the alpha frequency range during resting-state closed-eye conditions. EEG recordings from all three groups showed LRTC with power-law behavior. The presence of power-law scaling behavior in the LRTC of the EEG suggests that there are persistent temporal correlations in brain bioelectrical activity. This implies that there is a probability that minor variations in the EEG signal will be succeeded by minor variations and that major variations will be succeeded by major variations. The averaged scaling exponent across all brain regions considered was 0.82 for the control group, 0.84 for MDD, and 0.88 for OCD. The scaling exponent values are comparable to the results of previous EEG/MEG studies (Linkenkaer-Hansen et al., 2001, 2005; Nikulin and Brismar, 2004, 2005; Lee et al., 2007).

Previous studies by Lee et al. showed that depressed individuals had significantly higher scaling exponent values in F3, C3, T3, T4, and O1 channels in the broad EEG frequency range from 0.6 to 46 Hz compared to healthy controls (2007). Bachmann and colleagues obtained similar results, showing statistically significant differences in the LRTC of P3-Pz channels between healthy and depressive subjects (Bachmann et al., 2014). In our study, the disorder groups also showed a tendency to have higher fractal scaling exponents as compared to the control group, although there were no statistically significant differences between the MDD and control groups. This is consistent with previous studies by Linkenkaer-Hansen et al. (2005) who found no differences between depressed patients and healthy controls in the alpha band. Leistedt et al. (2007) found no difference in the broad frequency range of 0.5–25 Hz in men in sleep EEG. Hosseinifard et al. (2013) found no significant difference between depressed patients and controls in narrow frequency bands. Bornas et al. (2013) reported that no differences were found for scaling exponents at any brain location in any band between groups of subclinically depressed and non-depressed individuals. The potential reasons for the variation in results between studies that report differences in the control and depressed groups and those that do not include the examination of different frequency bands, the use of windows of different lengths, differences in the gender composition of the samples, and medication use.

We found significant differences between the control and OCD groups in the left frontal, middle frontal, right frontal, and left parietal occipital regions, with OCD scores being higher. In addition, OCD scaling exponent scores were higher than MDD scores in the middle frontal and right frontal regions. We cannot compare these findings to previous studies as, to our knowledge, no research has explored LRTC in individuals with OCD. Studies of nonlinear EEG characteristics in OCD are markedly restricted. For instance, it was discovered that EEG complexity (measured with approximate entropy) may be a useful biomarker for predicting treatment outcomes in OCD patients (Altuğlu et al., 2020). Furthermore, a group of scientists led by Yazdi-Ravandi, examined the complexity of information processing by fractal dimensions (FDs) and discovered that individuals with OCD exhibited higher FDs in the frontal regions across all frequency bands in comparison to healthy controls (Yazdi-Ravandi et al., 2021). These findings align well with our study. It is worth noting, however, that the study yielded significant results in the beta and lower gamma bands, whereas our research was focused on the alpha rhythm.

The question of how LRTCs relate to the cognitive and emotional domains has not been fully explored. In the emotional domain, there is evidence that large-scale exponents of broadband and theta-band oscillations are positively correlated with negative emotion regulation strategies and depression scores in subdepressed individuals, presumably leading in some cases to the development of depressive disorder (Bornas et al., 2013). In the present study, there were no significant correlations found between scaling exponent scores and symptoms of OCD or MDD. The level of depressive symptoms in both the OCD and MDD groups was comparable, indicating that despite the overlap in clinical symptoms, unique features of brain function can be detected in both MDD and OCD and in controls by means of DFA.

In the cognitive domain, psychiatric disorders are associated with cognitive decline, with depression found to impair attention, executive function, memory, and speed of information processing (Millan et al., 2012; McIntyre et al., 2015; Perini et al., 2019). Identifying specific areas associated with cognitive impairment in depression is challenging. Numerous studies have been conducted on this topic, highlighting the significance of extensive brain networks that include many structures, such as the hippocampus, amygdala, cingulate, fornix, insula, medial, and dorsolateral prefrontal cortex (e.g., Culpepper et al., 2017; Touron et al., 2022). A selective review of neurocognitive impairment in OCD by Suhas and Rao (2019) highlights the severity of executive dysfunction and nonverbal memory deficits, and the role of frontostriatal circuits in the neurobiology of OCD. Since cognitive abilities are known to be influenced by the dynamics of neural oscillations at many spatial scales and frequencies (Varela et al., 2001; Buzsáki and Draguhn, 2004), it is reasonable to assume that the observed differences between OCD and controls and OCD and MDD, which are almost exclusively concentrated in frontal regions, reflect to some extent alterations in cognitive functions. Much of the fMRI studies have indicated a role of the frontal cortex and its connections with other regions in OCD (Stern et al., 2012; Göttlich et al., 2014; Van Velzen et al., 2015). However, examining cognitive and emotional processes was not the purpose of the current study, and these assumptions are speculative in nature.

A more objective interpretation of the results obtained could be related to the fact that LRTCs are considered a measure of the excitation/inhibition balance in neural networks. Rebalancing of these processes may contribute to the symptoms of MDD and OCD disorders (Richter et al., 2012; Godfrey et al., 2018; Kang et al., 2019; Rodrigues Da Silva et al., 2022). Both OCD and MDD have been found to have abnormalities in the inhibitory and excitatory neurotransmitter systems (e.g., Pittenger et al., 2011; Godfrey et al., 2018), which we believe can be reflected in the change in LRTCs compared to the control group. It should be noted that recent studies on LRTC have already revealed its informative value in relation not only to affective disorders but also found increased DFA in neurodevelopmental disorders such as autism spectrum disorder (Bruining et al., 2020), STXBP1 syndrome (Houtman et al., 2021), and Rett syndrome (Sysoeva et al., 2023).

Most previous studies have focused on finding differences between the MDD/OCD and control groups separately, so further studies of differences in LRTC between these pathologies are needed to gain more insight. In order to explain the differences found in the frontal and parietal occipital regions, it would be useful to investigate not only the RS EEG but also the EEG during different cognitive tasks. In addition, it would be useful to study the characteristics of neurotransmitter systems functioning to confirm the hypothesis of impaired inhibition and excitation and to confirm the use of the LRTC metric as an indicator of imbalance in these processes. This information may be useful in differential diagnosis, given the frequent comorbidity of OCD and MDD and their often-overlapping clinical manifestations.

The limitations of the study include the inability to predict the influence of drug therapy on EEG of the subjects as well as the prevalence of female gender among the study participants. Further complicating the interpretation of the results, the level of depressive symptoms in OCD was comparable to that in MDD in our subsamples. Among other limitations, it is noteworthy that participants with MDD were significantly older than those with OCD and the control group. The study revealed that older adults (aged 55–72) showed a decrease in long-range correlations in their EEG signals during motor tasks when compared to younger adults (Pavlov et al., 2020). However, it is important to note that our sample included only 4 participants over the age of 55: 2 with MDD and 2 from the control group, and we also examined the resting state EEG without assigning any tasks. It is important to note that further studies should also control for general anxiety levels, as OCD and MDD may share features of high anxiety.

## Data availability statement

The raw data supporting the conclusions of this article will be made available by the authors, without undue reservation.

## Ethics statement

The study protocol was 105 approved by the ethical committee of the Institute of Higher Nervous Activity and Neurophysiology of the Russian Academy of Sciences (Ethics protocol: No. 2, 30 April 2021, Ethics protocol: No. 1, 25 February 2021). All participants signed a written informed consent.

## Author contributions

EP: Formal analysis, Funding acquisition, Methodology, Visualization, Writing – original draft, Writing – review & editing. OM: Conceptualization, Supervision, Writing – review & editing, Investigation. GP: Data curation, Investigation, Supervision, Writing – review & editing. GK: Data curation, Investigation, Writing – review & editing. OS: Conceptualization, Supervision, Writing – review & editing.
